# Microbial contamination and efficacy of disinfection procedures of companion robots in care homes

**DOI:** 10.1371/journal.pone.0237069

**Published:** 2020-08-26

**Authors:** Hannah Louise Bradwell, Christopher W. Johnson, John Lee, Rhona Winnington, Serge Thill, Ray B. Jones

**Affiliations:** 1 Faculty of Health, University of Plymouth, Plymouth, Devon, United Kingdom; 2 Department of Microbiology, Royal Cornwall Hospital, Truro, Cornwall, United Kingdom; 3 Department of Nursing, Auckland University of Technology, Auckland, New Zealand; 4 Donders Centre of Cognition, Radboud University, Nijmegen, The Netherlands; Universidade de Coimbra, PORTUGAL

## Abstract

**Background:**

Paro and other robot animals can improve wellbeing for older adults and people with dementia, through reducing depression, agitation and medication use. However, nursing and care staff we contacted expressed infection control concerns. Little related research has been published. We assessed (i) how microbiologically contaminated robot animals become during use by older people within a care home and (ii) efficacy of a cleaning procedure.

**Methods:**

This study had two stages. In stage one we assessed microbial load on eight robot animals after interaction with four care home residents, and again following cleaning by a researcher. Robot animals provided a range of shell-types, including fur, soft plastic, and solid plastic. Stage two involved a similar process with two robot animals, but a care staff member conducted cleaning. The cleaning process involved spraying with anti-bacterial product, brushing fur-type shells, followed by vigorous top-to-tail cleaning with anti-bacterial wipes on all shell types. Two samples were taken from each of eight robots in stage one and two robots in stage two (20 samples total). Samples were collected using contact plate stamping and evaluated using aerobic colony count and identification (gram stain, colony morphology, coagulase agglutination). Colony counts were measured by colony forming units per square centimetre (CFU/cm^2^).

**Results:**

Most robots acquired microbial loads well above an acceptable threshold of 2.5 CFU/cm^2^ following use. The bacteria identified were *micrococcus species*, *coagulase negative staphylococcus*, *diptheriods*, *aerobic spore bearers*, and *staphylococcus aureus*, all of which carry risk for human health. For all devices the CFU/cm^2^ reduced to well within accepted limits following cleaning by both researcher and care staff member.

**Conclusions:**

Companion robots will acquire significant levels of bacteria during normal use. The simple cleaning procedure detailed in this study reduced microbial load to acceptable levels in controlled experiments. Further work is needed in the field and to check the impact on the transmission of viruses.

## Introduction

Life expectancy is increasing worldwide [1], contributing towards an increasing demand on health and social care resources [2], because human function deteriorates with age [3, 4]. There is an identified need for research on maintaining wellbeing of older people [5], to assist declining numbers of professional care workers [1]. Improving wellbeing is essential for those in long term nursing facilities, who are vulnerable to feelings of isolation and loneliness [6], and those with dementia, a condition associated with changes referred to as behavioural and psychological symptoms of dementia (BPSD), and includes agitation, anxiety, depression, delusions and hallucinations [7]. BPSD can reduce wellbeing, but also increase care provider burden and distress [7, 8], hospitalisation and healthcare costs [7] and is associated with institutionalisation and medication use, including antipsychotics, which have serious side effects [8], including cardiovascular issues [9], and mortality [10]. Companion robots may provide a non-pharmacological psychosocial intervention to assist with these healthcare challenges.

A systematic review showed that there was a wealth of research available on the use of social robots, or companion robots in care and long term nursing homes [11], with various robots and interactive toys available [12, 13]. Much of the previous research focused on Paro the robot seal [14]. The benefits of interaction with Paro for older adults, including those with dementia, are reduced depression and agitation [15], more adaptive stress response [16], reduced loneliness [17], and reduced nursing staff stress [16, 18]. Paro may also reduce use of psychoactive and analgesic medications [19], and even lower blood pressure [20]. Nursing staff previously discussed perceptions of Paro, noting the usefulness for older people and potential social benefits, with the device aiding interpersonal relationships [21]. It should be noted, the aim of companion robots is to augment human care, rather than replace. Similar is true of robots used in other care contexts (for example children with autism) [22, 23], and support has been reported for the social mediation effect of such devices [17, 24].

However, little has been published on practical maintenance considerations of companion robot use. A review of benefits of and barriers to Paro implementation in care settings noted infection concerns as a key barrier [25]. The Health Protection Agency [26] provides guidance for community infection control nurses, health protection nurses, and care home staff including the decontamination of equipment, but little is known about how to do this for new technologies such as companion robots. We demonstrated Paro and other robot animals and toys to hundreds of people as part of the eHealth Productivity and Innovation in Cornwall and the Isles of Scilly (EPIC) project [27] in 2017–18 in Cornwall, including many nurses and care home staff, who frequently raised concerns of hygiene and infection control. We also found in other work [28] that relevant stakeholders expressed concerns regarding cleaning. The Department of Health and Social Care [29] suggests good infection control is imperative to ensure service users receive safe care. A previous large-scale randomised controlled trial of Paro in long-term care facilities described the employed hygiene protocol [30], including cleaning Paro after each use with disinfectant spray and wipes, and cleaning the storage box weekly. This reflects the cleaning procedure suggested by the Paro website [31]. However, research was lacking on the efficacy of such procedures, or any potential risk that companion robots pose for care home residents in terms of microbial transmission.

### Background

We are aware of only two studies on infection control and Paro [32], only one of which reported a cleaning procedure based on use of the robot on a UK National Health Service (NHS) dementia ward for 9 months [33]. Dodds et al. included a broad cleaning protocol discussing risk reduction measures and processes before, during and after use of Paro. Results suggested cleaning was successful based on Adenosine Triphosphate (ATP) luminometer readings of below 50 relative light units (RLU). The authors, however, acknowledged the limitations of the assessment method [34], as although it provided an estimation of surface cleanliness it is impossible to convert luminometric results to number of microorganisms [35].

Sygula-Cholewinska et al. [35] suggested many studies indicated that intracellular ATP levels vary so much between microbial taxa that tests of ATP should not be viewed as indicative of the presence of microbial pathogens. They suggested the method should not be commonly applied due to limitations such as low sensitivity of commercial luminometers for microbe detection, poor result reproducibility, and environmental factors influencing measurement outcomes [35]. A literature review by Health Protection Scotland [36] found most studies showed no correlation between ATP and microbial contamination. They concluded there was insufficient evidence to support using ATP as a marker of microbiological cleanliness.

The protocol described by Dodds et al. [33], therefore, has limited quantitative microbiological support, as noted by Rowson and colleagues [34]. Furthermore, the research was limited only to Paro, that is reported to have anti-bacterial fur [37], thus restricting generalisability of results to a wider selection of companion robots that do not generally have anti-microbial coverings. There was also no identification of microbes conducted, and samples were taken periodically over 9 months, rather than before and after cleaning [33]. Thus, no comparison was provided to demonstrate the impact of the cleaning on either microbial load or removal of specific microbes. There was, therefore, still a strong requirement for research using more valid and standardised methods [34], as well as a range of companion robot alternatives that do not have the anti-bacterial properties of Paro, to begin establishing a tested cleaning procedure for companion robots used by older adults.

Previous research investigating general cleaning efficacy includes work by Santos-Junior et al. [38], who sampled high-touch surfaces in a nursing ward before and after cleaning. They used ATP bioluminescence assay, aerobic colony counts (ACC), *staphylococcus aureus* colony count, and resistance to methicillin [38]. They collected 80 samples over four weeks, 40 before cleaning and 40 samples 10 minutes after cleaning to allow disinfectants to dry. The disinfectant used was NIPPO-BAC PLUS. They collected samples with contact plates containing tryptone soya agar with neutralizers. Results were analysed following incubation, and suggested only two of the five sites tested demonstrated significant decrease in RLU. ACC results showed that on two sites, microbial load was higher after cleaning and disinfection. They concluded the cleaning and disinfection process showed little effectiveness.

Kenters et al. [39] also tested cleaning efficacy, exploring effectiveness of various disinfectants, using a known positive method of contaminating tiles with a test solution of *clostridium difficile* strains. The authors compared wipes and sprays of various ingredients using colony count and ATP. Their results suggested that wipes performed better than sprays with the same active ingredient. Wipes including hydrogen peroxide (1.5%) demonstrated the highest bactericidal activity.

Woodland, Whitham, O’Neil & Otter [40], assessed colony counts on healthcare cubical curtains before and after cleaning. They used swabs to sample from high-touch areas of 20 curtains. Samples were incubated then colony-counts were conducted and micro-organisms were identified using gram stain and colony morphology. Colony counts increased slightly immediately after laundering before declining by 56% after one week, and the two most frequently present microorganisms were *coagulase negative staphylococcus* and *micrococcus species*. They suggested current laundry procedures may not be completely effective. A limitation, however, of this study was reliance on swabbing, which can create greater variation in sampling than more standardised methods such as contact plates [41].

Similar research on infection control for companion robots appears lacking, other than that of Dodds et al. [33]. Indeed, a literature review of hygiene for robotic animals in hospitals identified that related research focused only on children’s toys and dolls [42]. The authors concluded little is known about the hygienic application of robotic animals in the clinical setting [42]. Previous research investigating microbiological hazards on children’s toys and play equipment included Martínez-Bastidas et al. [43], who found interaction with play-park equipment influenced microbial presence on both children’s hands and toys. *E*.*coli* was predominant, but *staphylococcus aureus*, *klebsiella pneumonia*, *serratia*, *giardia lamblia* and *hepatitis A* were also found. The importance of these results is emphasised by other studies that suggested a chain of transmission of infection not only from person to person, but from fomites (objects) to people [44, 45]. Randle and Fleming [46] supported this concern, finding toys specifically can spread infection between children in healthcare settings.

Rowson and colleagues [34] discussed infection control concerns with Paro noting that soft-toy type shells are notoriously difficult to decontaminate, with no clear guidelines present on best practice. They also acknowledged the need for quantitative microbiological evidence on adequacy of any decontamination procedures, particularly when considering robot use with vulnerable older adults and those with dementia [34].

Older adults may be particularly vulnerable to health consequences when exposed to pathogens due to a decline in immune function with ageing [47]. Older people also have reduced levels of gastric acid, and consequently experience increased risk of developing infectious gastroenteritis [48]. Furthermore, older adults residing in care homes are at particular risk, due to concentration of high-risk individuals in the environment, and the susceptibility of this environment to spreading pathogens [49]. Infections in nursing home samples are associated with higher rates of morbidity and mortality, hospitalisation, and healthcare expense [48]. It is therefore important to establish if companion robots can transmit potentially harmful microorganisms between users and to assess efficacy of cleaning methods to allow safe use of companion robots in such settings. This paper therefore begins to contribute to the necessity noted by Scholten et al. [42], for research furthering our knowledge on robot animals and infection control.

Although Paro appears to be the most well researched companion animal robot [14], other interactive toys and robots are commercially available, such as the dinosaur Pleo, Miro, or the Joy for All cat and dog. Some of these cheaper devices have been used in previous research with older adults [12, 13]. We therefore included a range of commercially available toys and robots with potential for use with older adults. As Paro has been designed with anti-bacterial fur that can be washed with anti-bacterial products [37], our study provides a comparison with the surfaces of possible alternative robots. Our study thus has implications for: (i) the use of current companion robots in health and social care settings, (ii) the materials to be used in future robot design, (iii) cleaning procedures for robots and toys in care homes and similar contexts, either for real-world or research purposes.

## Method

### Setting

This investigation formed part of a collaborative action research project exploring use of companion robots and alternatives in care homes for older adults and people with dementia. Non-probabilistic convenience sampling was used to select two care homes as research sites. Both homes provide residential care for individuals with and without dementia. Four residents in each home volunteered to take part. In the first home, four females participated with a mean age of 86 (SD 14.84). In the second home, three females and one male participated, with a mean age of 90.75 (SD 4.09). The study also involved collaborating with a microbiology laboratory, which follows UKNEQAS [50] and LABQUALTY [51] for external quality assurance of bacterial identification, and is also UKAS accredited [52].

Ethical approval for this study was discussed and waived by the Faculty of Science and Engineering committee at the University of Plymouth, as data collection involved no human participants, older adults volunteered to assist in handling companion robots, as they are familiar with them for non-research purposes. A highly ethical approach was taken, with written consent gained from collaborators who were fully informed on research aims and potential implications. The Microbiology Investigation Criteria for Reporting Objectively (MICRO) checklist was used to guide the writing of this manuscript (S1 File), although not all points were deemed relevant to this study design [53].

### Design

**Fig 1:**
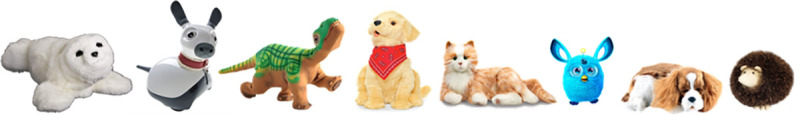
Eight robot and toy animals used in stage one. From left: Paro, Miro, Pleo rb, Joy for All dog, Joy for All cat, Furby Connect, Perfect Petzzz dog, Handmade Hedgehog.

Our study had two parts:

In stage one we investigated the microbial load on eight devices (Fig 1) following use, to establish contamination and infection risk. Tests were repeated after cleaning by the researcher, to assess efficacy of the procedure.

In stage two, we repeated this using only two animals (Joy for All dog and cat) with care staff themselves conducting the cleaning. The cat and dog had been present in the home for eight weeks, undergoing cleaning after each use by the care staff. Our procedure and materials were otherwise identical to stage one.

Both stages involved collection of environmental specimens during December 2018, in Cornwall, UK.

### Materials

#### Robots

A range of robots and alternatives were used (Fig 1).

Selection was based on current involvement in the larger project, and through providing a range of shell types and materials currently used on socially assistive robots (Table 1).

**Table 1:** Shell types of the robot animals and alternatives.

Animal	Shell Type	Fur Length (approx.)
Paro [54]	Anti-bacterial, anti-static soft fur (exact composition protected under intellectual property, but includes silver particles for anti-bacterial properties)	1cm
Miro [55]	Hard, smooth plastic	N/A
Pleo rb Dinosaur [56]	Soft textured plastic (SEBS thermoplastic elastomer)	N/A
Joy Dog [57]	Soft-toy fur (polyester, acrylic mix)	1cm
Joy Cat [58]	Soft-toy fur (polyester, acrylic mix)	2.5cm
Furby [59]	Soft-toy fur (polyester and acrylic mix) and hard smooth plastic	0.8cm
Perfect Petzzz Breathing Dog [60]	Soft-toy fur (100% polyester)	0.6cm
Knitted Hedgehog [61]	Soft toy fur (polyester and lurex mix)	2cm

#### Cleaning products

We used the following cleaning products for disinfection of the devices: (i) Sirafan Speed Disinfection Spray for Surfaces by Ecolab [62], and (ii) Super-Sani Germicidal Wipes by PDI [63]. Both companies currently supply disinfectants to health care providers. The use of both a spray and wipes was suggested by Moyle et al. [30] and the Paro user manual [31].

The PDI Super Sani-Cloths were selected as they are recommended for use in health care and medical settings to control cross contamination hazard, and also in the Paro cleaning instructions [31]. The wipes also allow for wiping of hard surfaces on devices, such as noses or eyes, and to allow the anti-bacterial product to be worked thoroughly into fur-type shells. Furthermore, research suggesting superiority of wipes over sprays despite similar composition [39]. The PDI company suggests these wipes are bactericidal, tuberculocidal and virudicidal, with broad coverage of microorganisms, including multi-drug resistant organisms [63]. The active ingredients include Isopropyl Alcohol, n-Alkyl dimethyl ethylbenzyl ammonium and chlorides. Although we have not tested for viruses here, this product also appears on the USA Environment Protection Agency List N of disinfectants meeting criterion for use against SARS-CoV-2 [64].

The Sirafan Speed Spray was suggested for trialling by contacts at Ecolab, due to the speed of disinfection and lack of rinse necessity, as rinsing is unfeasible for devices without removable skins. The disinfectant is suggested to be effective against bacterial, viral and fungal infections [62]. The active ingredients include Isopropyl Alcohol and 1-Propanol.

Products were selected for being more powerful than everyday disinfectants, due to the importance of intensifying disinfection on high-touch surfaces that could allow transmission of pathogens to service users [38]. Although both products are designed for hard surfaces, there is a lack of disinfectant products available specific to soft surfaces, and therefore currently available products may provide adequate substitutes. PDI and Ecolab currently supply to health and social care facilities, so the chosen products are easily accessible.

#### Agar plates

We used agar filled contact plates, supplied by Cherwell Laboratories. Irradiated tryptone soya agar was used, with four neutralisers to inactivate residual disinfectants. Plates were triple vent contact plates with a surface area of 25cm^2^. This type of agar is a general purpose nutrient agar currently used in environmental sampling, and is recommended for recovering a variety of microorganisms. Tryptone soya agar was used in previous research [38].

### Procedure

**Fig 2:**
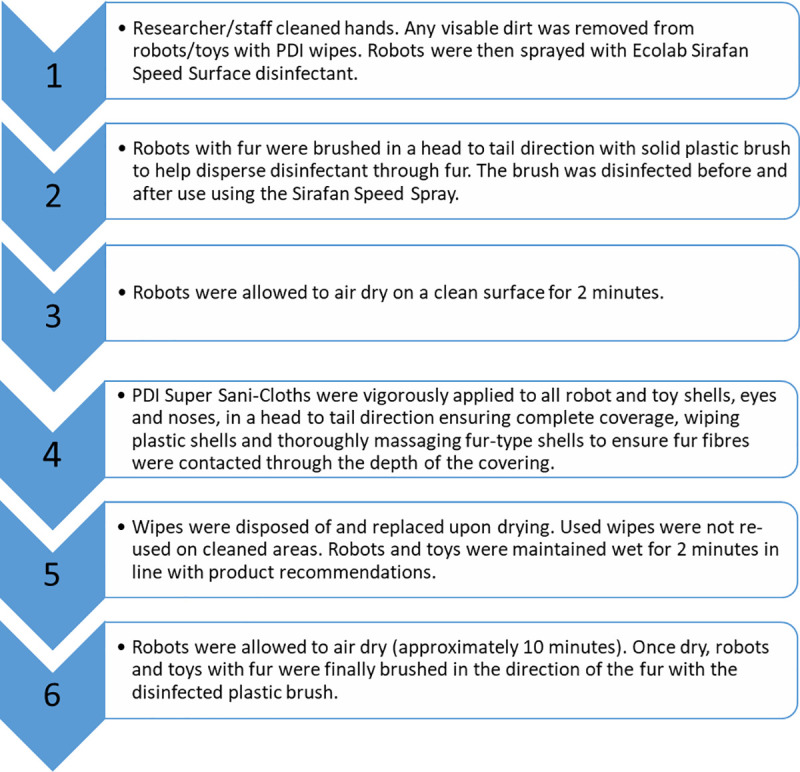
Cleaning procedure for use with socially assistive robots in care homes or other health and social care contexts.

The research was conducted in two care homes, reflecting the intended ‘real-world’ use of companion robots [11]. Devices were taken to two care homes providing residential care for older adults with and without dementia. Devices were cleaned using the described procedure (Fig 2) on site to minimise any influence of microbes collected during transportation.

The process of cleaning each robot or toy. The procedure took approximately six minutes, with additional drying time. This was applied to both soft-furry and hard-plastic shell types. Storage boxes and associated components such as chargers were also cleaned weekly using the same method.

Once cleaned, four care home residents interacted with the robots, in a group session reflective of real-world use and research practice [11, 14]. The four participants were invited to interact with each robot for five minutes with each robot receiving 20 minutes of interaction.

The researcher then sampled from the robots using contact agar plates to gain a measure of microbial load after use. Contact plates were applied to the sections of the robots most commonly touched based on review analysis of previous video recordings of 45 different care home residents interacting with each of the eight animals. This sampling of high-touch areas reflects previous methodology [38, 40]. The plate was in contact with the robot for 10 seconds, as in previous research [41].

The robots were cleaned again using the suggested hygiene procedure (Fig 2), then sampling was repeated to examine the efficacy of the cleaning method. This before and after cleaning sampling is suggested to be an established method of evaluating cleaning and disinfection practices [34, 65].

All sampling from the robots was conducted by the same researcher to standardise sample collection. Sixteen samples were collected in stage one, with each of the eight animals being sampled from once before cleaning and once after. Four samples were collected in stage two, with two animals being sampled before and after cleaning. Previous research by Woodland et al. [40], used swabs for testing microbial contamination of cubicle curtains in a health care setting, however the contact plate method allowed greater standardisation, and was used in previous research [38]. Sampling via swabbing requires two processes; sampling from the object itself and inoculation of the plate, while the contact plate method allows for inoculating of any bacteria directly from the object to the agar [41].

### Analysis

Samples were transported straight to the collaborating microbiology laboratory and incubated at 30–35°C for 5 days to grow any environmental organisms or enteric/pathogenic bacteria sampled from the animals. Colony counts were conducted at 48 and 120 hours, and CFU/cm^2^ calculated, providing an indicator of how ‘unclean’ robots become during standard care home use, and to assess the efficacy of the cleaning procedure, and initial comparisons of shell type. A threshold of ≤2.5 CFU/cm^2^ was considered acceptable, based on previous research [36, 38, 65]. In stage one, identification was conducted on colonies remaining after cleaning using gram stain, colony morphology and coagulase agglutination as in previous research [40]. This was to ascertain what microbes had remained following cleaning. In stage two, identification was conducted on micro-organisms present before cleaning, using the same methods. This allowed insight into microbes potentially transmitted on companion animals, and analysis of what microbes were removed during cleaning.

The datasets generated and analysed during the current study are available at the Open Science Framework using the following link: https://osf.io/4qud9/?view_only=183ae25f030a4e0b905a50286f99ca8c

## Results

### Stage one

Most of the devices gathered enough microbes during 20 minutes of standard use to have a microbial load above the acceptable threshold of 2.5 CFU/cm^2^ (Table 2).

**Table 2:** CFU/cm^2^ on each robot before cleaning and after cleaning at 48 and 120 hours incubated.

Animal	Before Cleaning		After Cleaning	
	48	120	48	120
Paro	3.20	3.20	0	0
Miro	0.04	1.08	0	0.64
Pleo	3.84	4.48	0.04	0.04
Joy for all Dog	8.96	9.60	0	0
Joy for all Cat	1.28	1.92	0	0
Furby	10.88	10.88	0.04	0.04
Perfect Petzzz Dog	17.28	19.20	0	0
Hedgehog	2.56	3.20	0.08	0.08

The Perfect Petzzz dog demonstrated particularly unacceptable levels, followed by the Furby and Joy for All dog. Only two of the animals remained within acceptable levels following use, the Joy for All cat and Miro. The post-cleaning CFU/cm^2^, however, demonstrates that regardless of material type, or previous microbial load, the described cleaning procedure effectively brought the CFU/cm^2^ on each animal down to well below acceptable levels, thus strongly supporting cleaning efficacy for bacterial contamination. Further to post-cleaning results being well within recommended limits, the remaining colonies following cleaning were identified as *aerobic spore-bearers* which are ubiquitous in the environment and pose relatively little risk.

### Stage two

The cleaning procedure was effective when carried out by care home staff (rather than the researcher). Using the benchmark of ≤2.5 CFU/cm^2^, it is clear microbial load on the animals was high following a group session, but that cleaning by a care staff member, following the standard procedure (Fig 2) removed microbes (Table 3).

**Table 3:** CFU/cm^2^ before cleaning, and after cleaning by a care staff member, at 48 hours and 120 hours incubation.

Animal	Before Cleaning		After Cleaning	
	48hr	120hr	48hr	120hr
Joy for All cat	24.32	29.44	0	0
Joy for All dog	5.76	10.24	0	0

Identification conducted on the samples taken before cleaning suggested the presence of *diptheriods*, *ASB*, *micrococcus species*, *coagulase negative staphylococcus* and *staphylococcus aureus*. Some of these bacteria can present a risk to human health [66, 67]. No gram-negative bacteria were present suggesting faecal contamination at time of sampling was unlikely. No colonies were present following cleaning.

## Discussion

The reported benefits of social robots have significant implications for health and social care, strongly supporting the use of such devices with older adults and individuals with dementia [13, 15–19]. Full implementation of companion robots however requires adequate protocols in place for safe and effective use. The concern of interest for our study was infection control, particularly for bacterial contamination. To the best of our knowledge, our study is the first of its kind in confirming, through initial empirical evidence, the strong requirement for adequate infection control procedures when using companion robots or toys in health and social care contexts. Previous research has suggested acceptable levels of aerobic colony counts are ≤2.5 CFU/cm^2^ [38]. Our results demonstrate that a single group session in a care home setting produced a microbial load higher than the accepted level on the majority of devices. These microbial loads identified the importance of adequate infection control, particularly with vulnerable people such as older adults [47], living in care homes [49]. This strengthens the need for validated cleaning techniques for use on socially assistive robots in health and social care settings, as noted by Rowson and colleagues [34].

The cleaning procedure we employed was informed both by previous research [30] and product recommendations [31], and our study provides initial empirical support for the efficacy of this cleaning procedure. The reduction in colonies to well below the recommended threshold following cleaning in both stage one and stage two suggests the cleaning procedure and products described are effective and feasible, and that cleanliness results are replicable by care staff. The procedure appeared similarly effective for both fur-type and hard-shell robots. The procedure described in our study therefore has implications for research and practice, providing a possible solution for implementation or research with companion robots and toys, where infection control is a concern, such as care homes. This research has also suggests that when employing a suitable cleaning procedure, more economical robots can be cleaned to the same infection control standard as Paro, who has an anti-bacterial covering [37].

The contact plate samples in the current study were taken from the areas of animals touched most frequently, based on video review of interactions during the wider project. Santos-Junior et al. [38] suggested previously that high-touch surfaces constitute most risk for transmission of microorganisms, therefore the risk of microbial contamination would have been greater had adequate cleaning not been undertaken. The identification of *staphylococcus aureus* also demonstrates the importance of adequate cleaning. While it is present in normal human flora of many healthy individuals, it can cause superficial and sometimes serious infections when allowed to enter the bloodstream or internal tissues [67], a significant burden of morbidity and mortality for older adults [68].

Preventing the transmission of *staphylococcus aureus* is clinically relevant for infection control purposes because of the potential for transmission of methicillin-resistant *staphylococcus aureus* (MRSA) within the healthcare setting. Microbes such as *staphylococcus aureus*, including MRSA, can be transmitted by direct contact or through fomites [67]. Objects such as robots and toys are fomites with potential to form vehicles of microbial transmission [44, 46], and therefore should be treated with adequate infection control procedures. Brodie, Biley and Shewring [69] previously discussed risks of live animals in health and social care, including an MRSA outbreak potentially contributed towards by a cat. The authors suggested improved hygiene as the principle measure in reducing disease transmission. Our results suggest that the cleaning procedure of the current study removed *staphylococcus aureus* due to the complete absence of colonies following cleaning. The remaining colonies in phase 1 were identified as Aerobic Spore Bearers and therefore again, further to being well below the recommended threshold, present very little risk.

Given the high colony counts seen before cleaning, we suggest that if companion robots are used in group sessions, members of the group should have hands cleaned both before and after robot use, to limit any microbial transmission. The importance of hand washing has, of course, also been emphasised to control the spread of viruses, particularly the SARS-COVID-19 virus [70]. Despite the limitations of the previous research by Dodds et al. [33], a number of important points were identified in their paper, including avoiding use of Paro with individuals with infections, or open wounds. We would suggest this advice also applies to the wider use of socially assistive robots in care homes and other health and social care contexts. The high colony counts seen in this study have further implications for other materials used in care homes likely to form vehicles of transmission, particularly with regard to group sessions where objects may be shared amongst residents.

One interesting and slightly anomalous result was that Miro grew very few colonies even when ‘unclean.’ It may be that Miro remained cleaner due to the solid plastic case, although we cannot draw firm conclusions with the limited number of samples we collected from plastic shells. Rowson and colleagues [34] noted the difficulties in decontaminating soft-toys, and perhaps hard-shells are more suitable for infection control purposes. Alternatively Miro may simply have been exposed to fewer microbes due to limited physical interaction with this device: while the care home residents were free to touch, hold, cuddle and interact with each robot as they wished, we observed that Miro was physically touched less than the alternatives (who received kisses and cuddles in addition to petting). This variation in interaction may also explain the differences seen in microbial load before cleaning between the different animals. We cannot easily generalise from individual devices to the materials from which they are made as the infection load will depend on both material and interaction behaviour.

The devices, once cleaned with the stated products, are not expected to cause skin irritation or pose health risks, if allowed to dry thoroughly before use. However, care should be taken to read full product information [62, 63], and inform residents and carers of the products used to check for any allergies or skin sensitivities. The cleaning products detailed can be flammable, and thus care should be taken with the items themselves, although the product evaporates and thus contact with and flammability of the disinfected animals should cause no additional issues. Cleaning of devices should be undertaken by staff, following precautions, and away from any care home residents, or health and social care service user, to minimise risk of direct exposure to disinfectant substances. Products should also be stored securely and COSHH (Control of Substances Hazardous to Health) assessments undertaken [71].

The range of devices included is a strength of our study, as the objects provided a range of shell types, from hard plastic to soft and furry. The previous research was conducted only with Paro [33], which has anti-bacterial fur properties [37]. The results of our study therefore have wider implications and better generalisation, although further research is required, with larger samples over longer periods in more natural settings, for firm conclusions on effectiveness (as opposed to efficacy) and comparison between shell types. The inclusion of hard-shelled robots such as Miro would suggest this cleaning procedure may also be applicable for a wider group of robots with potential for use in health and social care, such as humanoids like Pepper [72] or telepresence devices such as Giraff [73], although checks should be performed for any cautions provided by individual product companies.

Another strength of our study was the use of contact plates. Woodland et al. [40] relied on swabbing, which creates greater variation and allows less standardisation than contact plates [41]. Furthermore, we used aerobic colony counts. ATP luminometer measures had been used previously [33], which are reported to have considerable limitations [35], while the use of aerobic colony counts before and after cleaning is an established measure of cleaning efficacy [34, 65].

Finally, our study has some ecological validity, that is, the research was conducted in care homes, providing residential care for older adults, which reflects well the current intended use for such devices [11, 14]. The older adults interacted with the animal devices in group sessions, again reflecting current use of the devices in real-world and research contexts [11, 14]. The animals were cleaned on site, both by the researcher in stage one, and by a care staff member in stage two, furthering the generalisability of results to real-world situations.

A limitation of this study was the relatively small number of samples, with 20 samples collected and analysed in total, and only four samples acquired from plastic shell-types. While our study gives users of such companion robots confidence in their use further research could be conducted to statistically analyse any differences between shell types in the harbouring of microbes. This could inform shell selection for future robot design. We recommend further research in this area utilising larger numbers of samples, and repeated testing to allow statistical comparison. A larger study would also allow assessment of how effectively this cleaning procedure could be translated to a larger scale with a longer time frame, a limitation to this study. However, regardless of shell type, it appears from initial investigation that employment of an adequate cleaning procedure can bring microbial load well below acceptable limits for all shell-types considered in the current study. An implication of this finding is that currently available robots and toys without anti-bacterial coverings may provide alternatives to Paro without posing additional contamination hazards. Future research may also look to establish efficacy of alternate cleaning products, particularly for any availability of disinfectant specific to soft-surfaces.

Nursing staff have education and training on infection control of care equipment [74]. Our study provides evidence based guidance on how to control infection on this new addition, companion robots, to the care home environment. As noted by Rowson and colleagues [34], surfaces in hospitals can allow transmission of nosocomial pathogens. We encourage further research, using the cleaning procedure detailed in the current study and maintaining a range of social robot shell-types, providing known positive trials with specific nosocomial pathogens, to further enhance confidence in the procedures efficacy and applicability to wider health care contexts, such as hospitals.

### Further work

As identified, there is little other work exploring infection control with companion robots, and more work is certainly needed, particularly due to the limited number of samples collected in this study and requirement for further in situ testing with care staff. This preliminary study would suggest little difference between more affordable devices such as the Joy for All devices and Paro, with the anti-microbial covering [37], in any case, our additional work demonstrated limited appeal for Paro and Miro, as both lack characteristics appealing to older adults [28], meaning they are unlikely to be implemented and used as much. In contrast, we know that more affordable Joy for All cats and dogs are being implemented widely [75, 76]. Of priority therefore, in response to this widespread implementation, further testing should examine transmission of viruses further to bacteria. Given the high numbers of deaths in care homes as a result of the SARS-CoV-2 virus [77], further studies of both bacterial and viral infection control on robot companions are urgently needed.

In summary, our study provides a basis for further research in this area, and is highly relevant, due to considerable interest in use and implementation of companion robots in contexts such as care homes [11, 14, 15, 78], and due to the significance of any issues in infection control for this setting. Older adults are particularly vulnerable [47], as are individuals in care homes [49]. The implications of infection can be catastrophic, including mortality [48]. Rowson and colleagues [34] previously reported the need for evidence supporting adequacy of decontamination techniques for Paro and similar robotic animals, using established methods such as ACC before and after cleaning [34, 65]. Our study provides the initial step for such research.

## Conclusion

Companion robots hold significant potential for improving aspects of health and wellbeing for older adults. Numerous benefits have been reported, however research has been lacking on the important factor of infection control. We have demonstrated through colony counts and microbe identification that robots and toys can pose a bacterial infection control risk in health and social care contexts such as care homes. Our simple cleaning procedure has efficacy and gives some confidence that companion devices with a range of soft and hard shell types can be used relatively safely and that cheaper devices are no more risky than Paro. However, further research is needed both addressing viral infections and the effectiveness of our procedures in situ in the longer term.

## Supporting information

S1 File (DOCX)
