# Supplementary material for: Microbial contamination and efficacy of disinfection procedures of companion robots in care homes
Source: PLoS One. 2020 Aug 26;15(8):e0237069. doi: 10.1371/journal.pone.0237069 (PMC7449478; doi:10.1371/journal.pone.0237069)
Supplement: S1 File — (DOCX) [file pone.0237069.s001.docx]

**MICRO Checklist**

The following are checklist responses to the MICRO checklist, found at doi:10.1186/s12916-019-1301-1

Item Number: Response:

1. Types of specimen detailed within manuscript (pg 10)
2. Sampling period included (pg 10)
3. Sampling strategy described (pg 12, 13)
4. Described, environmental testing for bacterial load and identification (pg 9, 10)
5. Geographical setting described (pg 10)
6. Clinical setting described (pg 9)
7. N/A
8. Identification method described (pg 14, 15)
9. N/A no susceptibility testing for this study
10. N/A
11. N/A no antimicrobial testing
12. Included in manuscript (pg 9)
13. Included (pg 9)
14. N/A, study aim was overall bacterial load
15. Population included (pg 9)
16. N/A
17. N/A
18. N/A
19. N/A
20. N/A
